# Odorant cues linked to social immunity induce lateralized antenna stimulation in honey bees (*Apis mellifera* L.)

**DOI:** 10.1038/srep46171

**Published:** 2017-04-07

**Authors:** Alison McAfee, Troy F. Collins, Lufiani L. Madilao, Leonard J. Foster

**Affiliations:** 1 Department of Biochemistry & Molecular Biology and Michael Smith Laboratories, University of British Columbia, 2125 East Mall, Vancouver, British Colombia, V6T 1Z4, Canada; 2 Wine Research Center; Food, Nutrition and Health Building, University of British Columbia, 2205 East Mall, Vancouver, British Columbia, V6T 1Z4.

## Abstract

Hygienic behaviour (HB) is a social immunity trait in honey bees (*Apis mellifera* L.) whereby workers detect, uncap and remove unhealthy brood, improving disease resistance in the colony. This is clearly economically valuable; however, the molecular mechanism behind it is not well understood. The freeze-killed brood (FKB) assay is the conventional method of HB selection, so we compared odour profiles of FKB and live brood to find candidate HB-inducing odours. Surprisingly, we found that significantly more brood pheromone (β-ocimene) was released from FKB. β-ocimene abundance also positively correlated with HB, suggesting there could be a brood effect contributing to overall hygiene. Furthermore, we found that β-ocimene stimulated worker antennae in a dose-dependent manner, with the left antennae responding significantly stronger than right antennae in hygienic bees, but not in non-hygienic bees. Five other unidentifiable compounds were differentially emitted from FKB which could also be important for HB. We also compared odour profiles of *Varroa-*infested brood to healthy brood and found an overall interactive effect between developmental stage and infestation, but specific odours did not drive these differences. Overall, the data we present here is an important foundation on which to build our understanding the molecular mechanism behind this complex behaviour.

Honey bees (*Apis mellifera* L.) face many challenges, but disease is perhaps the most significant^1^ and hygienic behaviour (HB) is an important method of disease control^2^,^3^,^4^,^5^,^6^. Bees perform HB by detecting, uncapping and removing diseased brood from the colony to reduce pathogen load, thereby improving disease resistance and leading to higher survival rates when challenged with *Varroa destructor*, American foulbrood and chalkbrood^2^,^3^,^4^,^5^,^6^,^7^. The most well-established method of selecting for hygienic bees is the freeze-killed brood (FKB) assay, in which patches of brood are frozen with liquid nitrogen, returned to the hive and evaluated after 24 h. The HB score is defined as the fraction of dead pupae that have been detected and removed^4^,^7^. Colonies that perform well in this test also have improved outcomes when challenged with real diseases, allowing the FKB assay to be an effective tool for selective breeding^7^.

There is a large body of evidence suggesting that hygienic bees identify diseased brood through olfactory cues^8^,^9^,^10^,^11^,^12^,^13^,^14^ and that they are more sensitive to and better at discriminating between them^8^,^14^. The antennae, bees’ main olfactory organ^15^, have been shown to play a pivotal role in HB with multiple independent research groups identifying significantly differentially expressed antennal genes in hygienic *versus* non-hygienic bees, as well as strong antennal biomarkers for selective breeding^9^,^13^,^16^,^17^,^18^. Odorant binding protein (OBPs) aid odour detection and are consistently upregulated in hygienic bees’ antennae. However, relatively little is known about precisely what odours the bees are detecting. One study investigated the volatile odours emitted from chalkbrood-infected larvae^12^ and several focused on possible cues from *Varroa*-infested brood^19^,^20^,^21^,^22^,^23^, but none investigated how they compare to FKB (the main selective test for HB) and very few confirmed the biological activity of the compounds^12^,^19^. Furthermore, how infested brood odour profiles change with respect to pupa development (and associated growth of the mite family) is yet unknown. In this study, we use gas chromatography-coupled mass spectrometry (GC-MS) to find striking differences in compounds emitted from FKB and *Varroa*-infested brood across developmental stages. We also functionally validate the biological activity of candidate HB-inducing compounds by quantifying the strength with which they stimulate antennae of hygienic and non-hygienic bees, ultimately suggesting that brood pheromones and lateralization of olfactory sensitivity may be key features of HB.

## Results

### FKB-specific compounds

**Figure 1:**
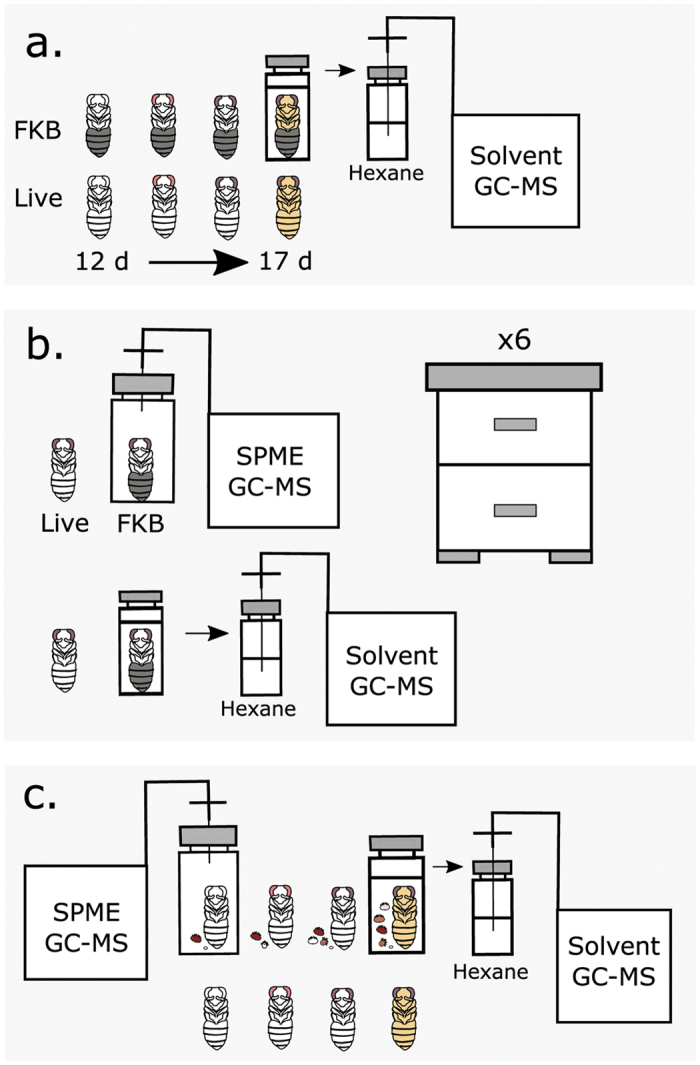
Experimental design schematics. (**a**) Cuticular hydrocarbon analysis. N = 3 for each developmental stage (white-eyed, pink-eyed, purple-eyed white body, purple-eyed tan body). FKB: Freeze-killed brood; GC-MS: gas chromatography mass spectrometry. (**b**) Cross-colony comparison of headspace volatiles and cuticular hydrocarbons. N = 3 for each colony. SPME: solid phase microextraction. (**c**) *Varroa*-infested brood headspace volatiles and cuticular hydrocarbons. Mites and their families were included in each sample. N = 3 for each developmental stage.

**Figure 2:**
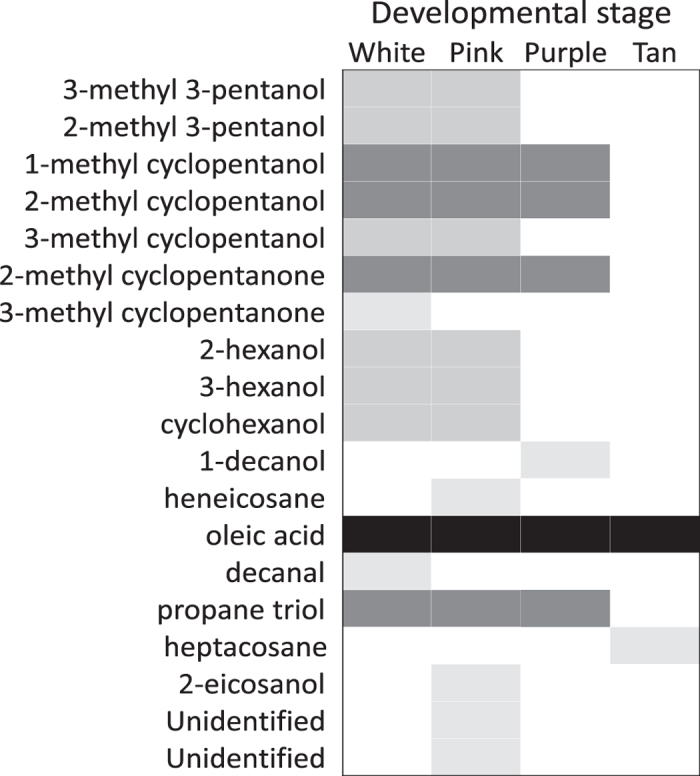
FKB-specific odour profiles vary across developmental stages. Cuticle compounds from live and freeze-killed white-eyed (12–13 d), pink-eyed (14–15 d), purple-eyed white body (16 d) and purple-eyed tan body (17–18 d) were analyzed using gas chromatography mass spectrometry (N = 3). Shaded boxes indicate compounds which had significantly different abundances in FKB compared to age-matched live brood. Compounds were identified by comparing mass spectra against a compound library. Only oleic acid was confirmed against a synthetic standard.

The FKB assay is thought to work equally well using any age of capped brood^7^, so we reasoned that candidate HB-inducing compounds should have consistently high abundances in dead relative to live brood across ages. To test this, we used GC-MS to compare the cuticle molecular profiles of 12 to 17 d old pupae at 1 to 2 d intervals (Fig. 1a). We found that indeed there were strong differences between dead and live brood (three-factor ANOVA; P < 0.000001; F = 597), which interacted significantly with developmental stage (P = 0.0000024; F = 9.72) and compound identity (P < 0.000001; F = 10.7). Young (12 to 15 d old) FKB tended to have more differentially emitted compounds compared to old (16 to 18 d) FKB (Fig. 2). While most of these compounds were age-specific, one compound, oleic acid, was consistently different across all ages. The identity of this compound was confirmed against a synthetic standard (Table 1; Supplementary Fig. S1).

**Figure 3:**
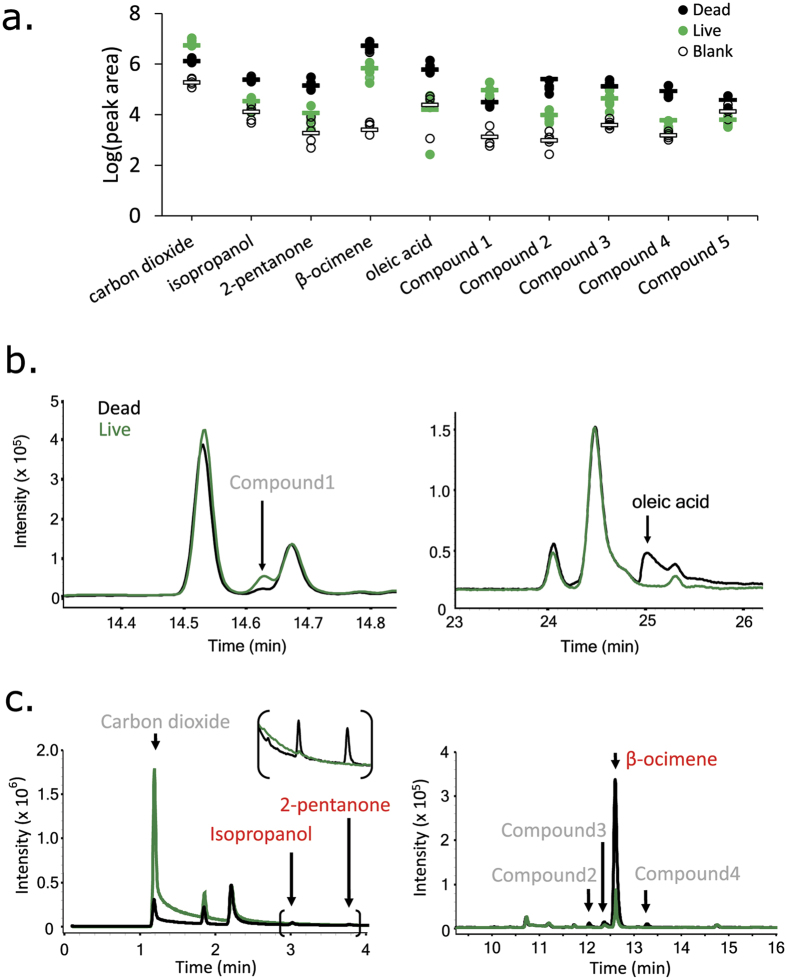
Cross-colony comparison of FKB and healthy brood odour profiles. (**a**) Ten compounds were significantly differentially expressed across colonies (n = 6; two-factor ANOVA; Tukey HSD; see Table 1 for p values). Compounds 1 to 4 were identified as 2-methyl tetradecane, α-thujene, α-pinene and 2,3-butanediol, respectively. Compound 5 (not displayed on chromatograms) could not be confidently matched to any spectra in the compound library. Bars represent averages. (**b**) Cuticle hexane wash and (**c**) Solid phase micro-extraction (SPME) example chromatograms covering the differentially emitted compounds. Bracketed region is enlarged for clarity.

We hypothesized that the compounds most likely to be HB-inducers should also be consistently differentially emitted from dead brood across diverse colonies. We compared the odour profiles of FKB to age-matched healthy pupae across six colonies located at three different apiaries. We found ten compounds that were consistently different between FKB and healthy pupae (Fig. 3a and b), although the identities of only four (isopropanol, 2-pentanone, β-ocimene and oleic acid) could be confirmed with synthetic standards (Table 1; Supplementary Fig. S1). For the five unknowns (Compounds 1 to 5), either the retention times of the synthetic standards did not match the peaks in the samples, making the identifications assigned by the spectral search algorithm unlikely, or the spectra could not be confidently matched to any in the comprehensive Wiley/NIST compound library. Of the ten compounds, nine were most abundant in the FKB headspace samples and only one was most abundant in live pupae. This peak had the highest volatility and a strong 44^+^ base-peak ion, which matches carbon dioxide and is consistent with active respiration. The carbon dioxide peak had above-background levels in the dead samples (although still significantly lower than in live samples), which is consistent with the decomposition expected to occur at warm temperatures.

### FKB odour strength is correlated with HB score

**Figure 4:**
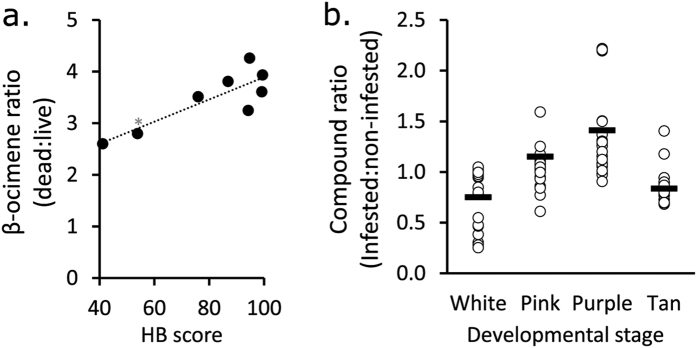
β-ocimene is a key compound in FKB, but not Varroa-infested brood. (**a**) Except where otherwise indicated, two rounds of hygienic testing were performed on eight different colonies. Out of all the significantly differentially emitted compounds, β-ocimene was the only one to significantly correlate with hygienic behaviour (Pearson correlation coefficient = 0.84, P = 0.0059; N = 3 within each colony. *This colony was scored based on one round of hygienic testing. (**b**) *Varroa*-infestation has a significant interacting effect (three-factor ANOVA; P = 0.000022; F = 8.34) on cuticle compound abundance, but specific compounds did not drive the effect (P = 0.99; F = 0.38).

It has been established that hygienic adult workers have superior olfactory sensitivity compared to non-hygienic bees^8^,^10^; however, the brood itself could also play a role in the behaviour^17^. Since a stronger odour should be easier for adult workers to detect and act upon, we hypothesized that brood from highly hygienic colonies may emit a stronger odour signal relative to healthy controls. In other words, there could be a brood effect contributing to overall hygiene. To test this, we correlated the dead:live ratio of each compound with HB score across eight different colonies. We found that only one compound was significantly correlated with the behaviour: β-ocimene (Fig. 4a; Pearson coefficient = 0.84; P = 0.0059; α = 0.0063; Bonferroni correction). Given that this compound is a familiar brood pheromone that is already known to increase worker visits to cells^24^, this is a remarkable result. Interestingly, β-ocimene was also consistently one of the most intense peaks observed in the chromatograms of dead brood (Fig. 3c), despite – to our knowledge – not being previously thought to be associated with HB.

### *Varroa* infestation interacts with developmental stage to alter cuticle profiles

To identify chemical cues associated with *Varroa* infestation, we compared odour profiles between infested and non-infested brood. *Varroa* mites reproduce inside the developing pupa’s comb cell, forming a whole family (including the foundress, eggs, protonymphs, deutonymphs and adult males) over time (Fig. 1c). We included four sequential developmental stages (white-eyed, pink-eyed, purple-eyed white body and purple-eyed tan body) and included the mite families with the pupae in the analysis. We did not find any significant effect of infestation in the headspace volatile profile (three-factor ANOVA; P = 0.46; F = 0.56); however, analyzing the cuticle profile showed that while infestation had no effect on its own (three factor ANOVA, P = 0.28, F = 1.15), it significantly interacts with developmental stage (Fig. 4b; P = 0.000022; F = 8.34). The overall trend was for infested brood to produce higher levels of compounds relative to healthy brood in age-matched adjacent cells, but no individual compounds drove this effect.

### Electroantennography shows lateralization of olfactory sensitivity in hygienic bees

**Figure 5:**
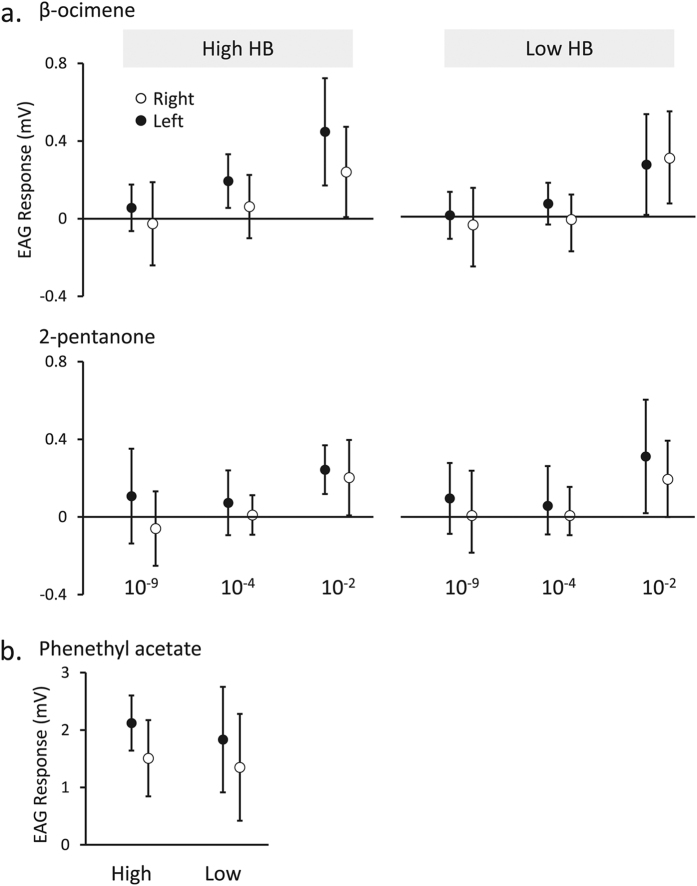
Antenna stimulations by candidate HB-inducing compounds. Electroantennography (EAG) was used to quantify antenna responses to odour stimuli. 2-pentanone and β-ocimene doses were applied at three dilutions (10^−9^, 10^−4^ and 10^−2^ v/v). The response to 2-pentanone was dose-dependent and lateralized but did not depend on HB (three-factor ANOVA). The response to β-ocimene was also dose-dependent and lateralized with a significant interactive effect between HB and dose as well as HB and side (three factor ANOVA). Phenethyl acetate, a known HB-inducing compound, was applied at one dose (10^−9^ v/v). A significant interactive effect was observed between HB and side (two-factor ANOVA). See Table 2 for statistical information and Table 3 for biological replicate numbers. Error bars represent standard deviation.

We investigated the biological activity of isopropanol, 2-pentanone, β-ocimene and oleic acid using electroantennography (EAG) to quantify antennal nerve depolarizations of hygienic and non-hygienic bees in response to stimuli (Fig. 5). Of all the compounds, only 2-pentanone and β-ocimene showed dose-dependent responses (three-factor ANOVA; see Table 2 for summary statistics). For β-ocimene, we also found significant interactive effects between dose and HB as well as HB and antenna side. Notably, the left antenna of hygienic bees produced the strongest EAG signal overall – significantly higher than the right antennae – whereas non-hygienic bees did not display this effect. This is counterintuitive, since right antennae have a higher proportion of olfactory sensilla^25^ and foragers give stronger EAG responses to (-)-linalool and isoamyl acetate (alarm pheromone) through their right antenna^26^. However, we confirmed that the same left-biased lateralization holds true for another known HB-inducing compound, phenethyl acetate^12^ (isolated from chalkbrood; Fig. 5; Table 2). Surprisingly, oleic acid appeared not to stimulate bee antennae at all, possibly because of its low volatility at room temperature, and isopropanol stimulations produced no significant differences (Supplementary Fig. S2).

A well-known phenomenon in olfactory perception is the synergistic effect of odorant mixtures^27^. That is, mixtures can be perceived not as the sum of their parts, but as if they are entirely new odours; however, this is rarely observed in honey bees^28^,^29^,^30^. To test if the four odours could lead to stronger EAG signals by stimulating antennae synergistically, we produced equivolume mixtures (1% total in ethanol) of all possible combinations of isopropanol, 2-pentanone, β-ocimene and oleic acid and used these to perform EAG on left antennae of hygienic bees. As expected, none of the odour combinations induced greater antenna stimulations than β-ocimene alone (the strongest stimulator; Supplementary Fig. S3).

### No proteomic differences were observed between left and right antennae

To determine a potential mechanism for lateralization of antenna stimulation at the gene expression level, we performed quantitative proteomics on left and right antennae of nurse bees from five hygienic colonies. Despite identifying 1,845 proteins (13,128 peptides) at 1% FDR, none of them were differentially expressed (Supplementary Fig. S4). Interestingly, 230 of the identified proteins are ones that were discarded from the first Official Gene Set (OGSv1.0), apparently in error. We described this phenomenon previously^31^ and this finding offers secondary confirmation. A further 15 proteins are new sequences which we identified in the same previous proteogenomic effort.

## Discussion

Overall, our experimental findings point to emerging mechanistic patterns regarding HB. We found that a well-known brood pheromone, β-ocimene, was strongly emitted from FKB and this pattern positively correlates with HB score. We also identified one compound, oleic acid, which was consistently released in higher amounts in FKB, not only across colonies but also across developmental stages. Finally, we functionally validated these compounds using electroantennography and show that lateralization of antennal response is strongly associated with HB, but we could not identify associated proteomic changes. Unlike the FKB, we found that *Varroa*-infestation causes subtle but significant changes to the overall cuticle compound profile, although no individual compounds emerged as drivers. This may in part explain why trait selection for *Varroa*-sensitive hygiene, a specialized form of HB, requires more rigorous selection techniques.

The clear majority of differentially emitted compounds were more abundant in dead pupae than in live ones. This is intuitive, since HB-triggering compounds should give a more reliable and specific signal to the bees if dead:live discrimination is based on their presence, rather than absence. Interestingly, three different likely terpene peaks were identified (based on a MS2 base peak of 93.0 m/z and spectral matches to other terpenes; Table 1) but of these, only β-ocimene could be confidently confirmed. These unidentified compounds could still certainly be biologically relevant to HB, but further work is required to identify them.

Oleic acid – an omega-9 monounsaturated fatty acid – was emitted more strongly in FKB compared to live brood of all ages tested in this study. Intriguingly, oleic acid has been implicated as a mechanistic agent for HB in other social insects^32^,^33^. For example, Wilson *et al*.^33^ found that applying oleic acid to otherwise mobile and healthy ants induced other ants to transport them to their refuse area. Oleic acid also binds strongly to odorant binding protein 18, which significantly correlates with HB in honey bees^9^ and is currently being used for marker-assisted selective HB breeding^18^. Finally, it has been shown previously that *Varroa*-parasitized brood (which can also trigger HB) emits more oleic acid compared to healthy brood; however, at the time of that study it was not identified as a discriminating compound^20^, and we could not replicate these results in our analyses of *Varroa*-infested pupae. Despite this, oleic acid did not elicit a strong EAG signal, possibly because of its low volatility (oleic acid boiling point: 360 °C; 2-pentanone was the next highest at 101 °C).

β-ocimene is a well-known brood pheromone which plays multiple roles in regulating worker behaviour and anatomy^24^,^34^,^35^,^36^,^37^,^38^. Young larvae normally release β-ocimene to stimulate workers to feed them^24^, with levels tapering off with age. β-ocimene also plays a role in regulating forager activity^38^ and inhibits worker ovary development^36^, but, to our knowledge, its release has not been previously associated with the FKB assay or HB. It seems unusual that dead bees would emit more of a brood pheromone than live bees, but it could be that a normally tightly controlled pheromone release mechanism breaks down as membranes become more permeable after freezing. β-ocimene was also the tallest peak in the chromatograms and was the only compound to elicit both dose-dependent and HB-dependent EAG responses (along with the known HB-inducing compound: phenethyl acetate; Fig. 5, Table 2). This finding is intriguing for two reasons: 1) β-ocimene has previously been shown to increase the frequency of worker visits to brood^24^ and 2) an independent study found that a different brood pheromone (brood ester pheromone; BEP) was also significantly more abundant in parasitized brood^23^. By increasing worker visits to brood cells that should otherwise not require attendance, β-ocimene may attract the attention of bees that can perform HB. As the second brood pheromone implicated in HB, these results may indicate a broader pattern of HB dependence on brood pheromones. Furthermore, Mondet *et al*.^23^ suggest that BEP contributes to detection of *Varroa*-infested pupae by signaling developmental delay – this mechanism is consistent with our own observations, since more β-ocimene is emitted from larvae compared to pupae^35^.

Further stimulating our interest in this compound, we also found that the ratio of β-ocimene emitted from FKB to live pupae significantly positively correlates with HB itself. This suggests that there may be a brood effect contributing to HB scores, in addition to olfactory sensitivity of adult workers, even though previously this was not thought to be the case. In an early foundational paper, Spivak and Downey^7^ found no brood effect when they performed hygienic tests using reciprocally donated brood; however, brood age was not controlled during these tests. In the same study, they established that brood age had a significant effect on non-hygienic colonies but not on hygienic colonies, with non-hygienic colonies performing significantly better on the FKB test when young brood (capped larvae and prepupae) was used compared to older pupae. The confounding factors may have simply erased potential brood effect patterns. Interestingly, β-ocimene is also present in far higher amounts in larvae compared to pupae and the larva cuticle is more delicate, so it is possible that disruption of the larval membranes by freeze-killing leads to an even larger proportion of β-ocimene emitted. It would be worthwhile to examine this effect across more diverse sources of high and low HB colonies to determine if it is a ubiquitous theme, or if there is a distribution of colonies that achieve hygiene through a brood effect, worker olfaction, or a mixture of the two.

This is not the first time that a brood effect has been suggested: Parker *et al*.^17^ found significant differences in the larval cuticle proteome between high and low VSH bees (a specialized form of HB targeting *Varroa* mites) and suggested that this may lead the brood to emit different chemical cues. This data, together with our own, suggests that HB could be dependent on two interacting factors – the strength of brood odour and the workers’ limit of odour detection – rather than the adult workers’ olfactory sensitivity alone.

The left-biased EAG response lateralization is intriguing. Lateralization in bees is not new: Rogers *et al*.^39^ have shown that bees are more likely to interact aggressively when communicating *via* their left antenna, whereas they have preferentially positive encounters when interacting *via* their right antenna. Interestingly, Rogers and Vallortigara^40^ found that bees performed better at long term memory recall tasks when stimulated *via* their left antennae, but not their right. We did not acquire data on the higher order processing of odours, but these studies create a precedent for antenna lateralization as it relates to behaviour. It is curious that despite having more olfactory sensilla on the right antenna^25^, the left elicits a stronger EAG signal for FKB and chalkbrood compounds. One possible explanation is that the olfactory sensilla that do exist on the left antenna house olfactory receptor neurons that are specifically tuned to particular odours.

Given the apparent lateralization of response observed here and electron micrographs showing more olfactory sensilla on the right antennae of worker bees^25^, we expected to also find differences in protein expression between left and right antennae of hygienic bees (Supplementary Fig. S4). The lack of any detectable difference indicates that we either did not penetrate deep enough into the proteome or that the contribution of differential expression in different sensilla is small compared to the overall expression of the relevant proteins. In the future, our proteomics analysis could be improved by performing sample fractionation to increase depth.

When we compared odour profiles of *Varroa*-parasitized pupae to healthy pupae across four developmental stages, we found a significant interaction between parasitization and developmental stage but no individual compounds drove this effect (Fig. 4). This could be because VSH is a specialized form of HB^41^ and this specialization is required because the differences between infested and non-infested brood are subtler than for dead and live brood. Indeed, one strategy for mites to evade detection in the colony is to adapt their own cuticle hydrocarbon profile to mimic its host^42^. Another explanation is that since the healthy control brood was pulled from cells immediately adjacent to the infested pupa, it could be that *Varroa*-associated compounds transferred through the thin wax wall to the healthy pupae, diminishing the observable differences. However, we still believe that this was the appropriate comparison, since hygienic bees must be able to discriminate between neighbouring healthy and diseased states. Finally, it could also be that key differentially emitted compounds do exist, but we were unable to detect them with our extraction method or our sample size.

Mondet *et al*.^23^ were recently able to find *Varroa*-specific compounds by analyzing solvent extracts of crushed infested pupae, although it is not clear that compounds measured in this way would be detectable by bees performing HB. The hexane extraction and SPME used here are suitable for capturing non-polar compounds with relatively high volatility but it could be that the superior olfactory sensitivity of hygienic bees allows them to detect some polar, non-volatile compounds. Indeed, oleic acid (a carboxylic acid) is one of our most confident HB-inducing candidates but it was among the last to elute in our GC-MS analysis of hexane extracts; more polar compounds would likely become trapped in the GC-MS inlet or not be miscible in hexane at all. Notably, Mondet *et al*. also observed that P5 pupae (roughly equivalent to our purple-eyed white-body stage) are targeted most frequently for VSH, following remarkably the same trend as overall compound abundance in infested pupae as displayed in Fig. 4b. This points to the possibility that VSH bees are either a) more sensitive to a specific compound found in the milieu of more abundant compounds emitted from *Varroa*-infested brood or b) VSH bees are broadly more sensitive to a suite of compounds associated with infestation.

## Conclusion

The work presented here furthers our understanding of HB and the underlying mechanism. Interestingly, this is now the second study to implicate a known brood pheromone (β-ocimene, in this case) as a mechanistic agent for HB. We found that hygienic bees, but not non-hygienic bees, elicit a dose-dependent lateralized olfactory response to β-ocimene. Furthermore, it is already known that odorant binding protein 18, which is thought to aid in odour detection, positively correlates with HB and here we show that one of its strongest known ligands (oleic acid) is indeed abundantly emitted from dead brood. This compound is also known to induce HB in other social insects, suggesting that the mechanism for HB is evolutionarily conserved. The odour profiles of *Varroa*-infested brood showed a significant interaction between infestation and developmental stage, and this subtlety is consistent with VSH being a specialized form of HB. Further experiments are needed to confirm the identities of the five unknown significant compounds since they may still be biologically relevant.

## Materials and Methods

### Honey bee colonies and hygienic testing

Honey bee colonies were kept at three separate locations in Greater Vancouver, BC, Canada. Colonies were scored for HB using the FKB assay as previously described^7^. All testing and sampling was conducted during the summer of 2016.

### FKB GC-MS sample collection

Honey bee pupae with no visible signs of disease were collected from colonies by carefully uncapping cells and removing pupae with clean stainless steel forceps. Age was determined based on eye and cuticle pigment using the following relationships: white-eyed = 12–13 d, pink-eyed = 14–15 d, purple-eyed white body = 16 d and purple-eyed tan body = 17–18 d. From bee to bee, eye and cuticle pigment was matched exactly so that each bee in each age group was at the same developmental stage. Pupae were placed in clean glass vials, removing any wax debris and avoiding abrasions or cuticle indentations. Freeze-killed samples were placed at −80 °C (15 min) then placed in a humid 33 °C incubator (24 h), while live samples were placed directly into the same incubator. After the 15 min freeze, all pupae were completely solid and brittle so there is no doubt that they were mortally frozen.

Compounds were extracted for low resolution GC-MS analysis by two different methods: solvent extraction and solid phase micro-extraction (SPME). For analyzing cuticular compounds across developmental stages (white-eyed, pink-eyed, purple-eyed white body and purple-eyed tan body; n = 3), extracts were prepared by washing whole pupae with 300 μl HPLC-grade hexane for 5 min with gentle agitation. Hexane extracts were transferred to a clean vial and immediately stored at −80 °C until GC-MS analysis. For the cross-colony analysis (N = 3 per colony, n = 6 colonies), compounds were extracted only from purple-eyed white body pupae using the method above as well as by sealing individual freeze-killed and live pupae in 10 mL glass vials (Supelco) and incubating at 33 °C (24 h) for SPME analysis. We confirmed that 10 mL of air is enough for one bee to survive for this time by performing the same procedure for late-stage pupae, which were still actively moving after being sealed for 24 h.

One μL of each hexane extract was analyzed by GC-MS (Agilent 6890 N/5975 C Inert XL MSD) using a DB-wax column (J&W 122–7032) and a 30 min gradient from 50 °C to 230 °C. The back inlet (pulsed splitless) was at 250 °C and 6.24 psi with a 53.5 mL/min flow rate (He gas) connected to the analytical column (30 m, 250 μm ID). The instrument was set to scan from 40–300 m/z. The MS source and quadrupole were maintained at 230 °C and 150 °C, respectively.

Headspace volatiles were sampled using solid phase micro-extraction (SPME) and analyzed by GC-MS (Agilent 7890 A/5975 C Inert XL MSD) using a 45 min gradient and the same column model as above. We used a 50/30 μm DVB/CAR/PDMS stableflex SPME fiber and sampling details were: 40 °C incubation, 3 s agitation at 500 rpm, 600 s extraction time and 300 s desorption time. The oven settings were: 35 °C (stable; 4 min), then 25 °C/min (5 min) and a 2:1 split ratio. The inlet temperature was 250 °C and MS acquisition parameters were the same as above except that the lower mass limit was 33 m/z.

### *Varroa* destructor GC-MS sample collection

For ease of sampling, mite-infested brood were concentrated on a single frame by caging the queen in a single-frame excluder and transplanting all other open brood into a temporary ‘incubator’ colony. This left only the single frame of brood suitable for mite infestation, effectively concentrating the phoretic mites looking for brood cells in that colony to one location. After 10 d, the brood was returned from the incubator colony and the queen was released. Following this, pupae were sampled by the same methods as above and only pupae with a single foundress mite were chosen. The accompanying mite family (including foundresses, deutonymphs, protonymphs and eggs; Fig. 1c) was transferred to the same glass vial as the pupa using a soft paintbrush. Adjacent, age-matched non-infested sister pupae with no visible signs of disease were collected as controls.

### GC-MS data analysis

GC-MS data was analyzed using Mass Hunter Qualitative Analysis software (vB.06.00). Chromatogram peaks were first smoothed using the default algorithm and then manually integrated to ensure consistent baselines between replicates. To compare odour profiles of FKB to healthy pupae across developmental stages, peak areas were exported to Excel (2013) where they were log_10_ transformed and groups (developmental stage, freezing, compound type) were compared using three-factor ANOVA (Excel), followed by a Tukey HSD *post-hoc* test to identify the specific differentially emitted compounds. We did not test the data for normality, but the ANOVA is generally tolerant to non-normal data and/or low replication. The same process was used to analyze FKB changes across colonies except that a two-factor ANOVA was employed since this only involved a single developmental stage (purple-eyed white body pupae). The effect of *Varroa*-infestation was also examined using a three-factor ANOVA (developmental stage, infestation, compound). In all cases, compound identities were determined by searching spectra against the Wiley Chemical Compound Library (W9N08.L) in Mass Hunter.

To determine if any of the significantly differentially emitted compounds – including those with unassigned identities – correlated with colony HB score, we calculated the dead:live ratio (not log transformed), then the Pearson correlation for each compound. We did not attempt to correlate the carbon dioxide abundance, since this is an artifact of respiration, nor did we include compound 5, which was significantly higher in the FKB compared to live, but not to the empty background. In total, 8 compounds were correlated. To account for multiple hypothesis testing, significance was determined by comparing P-values against the Bonferonni-corrected α (0.05/8 = 0.0063).

### Antenna preparation for electroantennography

Bees for electroantennography (EAG) were collected across three colonies with high HB scores and three with low HB scores. Since bees perform HB best when they are two to three weeks old^43^, we marked emerging bees with a paint pen and returned them to the hive for 14 days, then EAG data was acquired for up to one week. Antennae were excised and both ends were trimmed with a scalpel, randomizing whether right or left antennae were excised first. Trimmed antennae were then attached to glass capillary reference and recording electrodes filled with insect saline solution (210 mM NaCl, 3.1 mM KCl, 10 mM CaCl_2_, 2.1 mM NaCO_3_, 0.1 NaH_2_PO_4_) as previously described^44^. EAG responses were recorded on the EAD program of a Syntech™ IDAC-4 signal acquisition unit. The low cutoff was set at 0.1 Hz, high cutoff at 10 Hz, external amplifier set to 1. Humidified, charcoal filtered air was passed continuously over the antenna via a Syntech CS-55 stimulus controller, also serving as a carrier for odour-filled pulses. Odorants were dispensed onto 1 cm^2^ No. 1 Whatman filter paper, allowing the solvent to evaporate for 30 s before being inserted into a glass Pasteur pipette. Odorant pulses were passed through the Pasteur pipette to the antenna for 1 s, and 0.5–1 minute was allowed between each presentation of an odour for the antenna to return to baseline activity. Each antenna was stimulated with a series of three dilutions (10^−9^, 10^−4^ and 10^−2^v/v in ethanol) each of isopropanol, 2-pentanone, β-ocimene and oleic acid (all from Sigma or Fisher; >90% purity). Phenethyl acetate, a known HB-inducing compound isolated from chalkbrood^12^, was used as a positive control. All possible equivolume combinations of the four candidate compounds were also tested at a 10^−2^ (1%) dilution to test for synergistic effects of mixtures.

Even though antennae were conditioned with humidified air throughout the recordings, EAG signal decay was still evident even for stimuli of solvent alone over time. Therefore, each antenna was subject to intermittent solvent stimulations throughout the recordings to mathematically interpolate the background solvent stimulus. The quality cut-off for the solvent curve fit was R^2^ > 0.8: traces which did not meet this criterion were discarded. The final replicate numbers included in subsequent analyses are show in Table 3. Since the number of surviving traces varied, in total we acquired between 10 and 15 biological replicates in each experimental group (left vs. right; high HB vs. low HB). Finally, the interpolated solvent amplitude was then subtracted from the solvent + odour stimulations, resulting in the mV value that can be attributed to the odour alone. The amplitudes of our recordings are consistent with other similar studies in bees^8^,^11^,^40^. Statistical analyses were conducted using a three-factor ANOVA (dose, HB and antenna side) and a Tukey HSD post-hoc test.

### Antenna protein extraction and proteomic analysis

Thirty to forty bees on open brood frames were collected from five highly hygienic colonies (n = 5; all with FKB scores >94%; Table 4). Bees were anesthetized with carbon dioxide and their antennae dissected on ice followed by homogenization (Precellys 24; Bertin instruments) with ceramic beads (lysis buffer: 6 M guanidinium chloride with 10 mM TCEP, 100 mM Tris (pH 8.5), 40 mM chloroacetamide). The homogenizer was set to 6,400 M/s for 30 s × 3 (1 min on ice in between). Lysate was transferred to a new tube and debris was pelleted (16,000 rpm, 15 min, 4 °C), followed by acetone precipitation as previously described^45^. Dried protein pellets were resuspended in 50 mM ammonium bicarbonate buffer (1% sodium deoxycholate) and protein concentration was determined using a bicinchoninic assay (Pierce). Protein was reduced, alkylated, digested and analyzed on an LC-ESI-MSMS system (Easy nLC-1000 coupled to a Bruker Impact II mass spectrometer) as described in our previous publication^31^, except we loaded 2.5 μg (based on protein quantitation), the LC gradient was 165 min and MS/MS frequency was set to 18 Hz (see embedded microTOFQImpactAcquisition.method files within PXD005242 for further details).

Proteomics data was searched using MaxQuant (v1.5.5.30) and processed using Perseus (v1.5.5.3). All data was deposited to ProteomeXchange (PXD005242). All MaxQuant search parameters were left as default except: deamidation (NQ) was added as a variable modification, “match between runs”, “label-free quantification” and “re-quantification” options were enabled and “min ratio count” was set to 1. Briefly, reverse hits, proteins “only identified by site” and contaminants were removed followed by filtering for proteins identified in four or more colonies. Data was then Log2 transformed and missing values were imputed before comparing left and right antennae using a two-group comparison in Perseus.

## Additional Information

**How to cite this article**: McAfee, A. *et al*. Odorant cues linked to social immunity induce lateralized antenna stimulation in honey bees (*Apis mellifera* L.). *Sci. Rep.* **7**, 46171; doi: 10.1038/srep46171 (2017).

**Publisher's note:** Springer Nature remains neutral with regard to jurisdictional claims in published maps and institutional affiliations.

## Supplementary Material

Supplementary Information

## Figures and Tables

**Table 1:** Differentially emitted compound identifications (experiment diagrammed in Fig. 1b)^1^.

Compound^2^	Base peak (m/z)	Score^3^	Sample RT (min)	Standard RT (min)	Identification accuracy^4^	P-value
Isopropanol	44.99	90.6	3.0	3.0	High	2.2E-23
2-pentanone	43.01	91.4	3.8	3.9	High	3.4E-23
*E-*β-ocimene	93.00	94.2	12.6	12.7	High	2.3E-33
Oleic acid	55.07	72.6	25.0	25.1	High	1.8E-70
Compound 1 (α-thujene)^3^	93.00	82.3	12.1	5.5	Low	9.5E-10
Compound 2 (α-pinene)	93.00	94.4	12.4	5.6	Low	2.5E-12
Compound 3 (2,3-butanediol)	42.98	79.2	13.3	22.4	Low	1.2E-13
Compound 4 (2-methyl tetradecane)	57.09	83.5	14.7	N/A	Low	1.6E-45

^1^Corresponding data is shown in Fig. 3.

^2^Bracketed compound names represent the proposed Mass Hunter matches.

^3^Mass Hunter Qualitative Analysis v.B.06.00.

^4^Based on comparison to synthetic standards.

**Table 2:** Summary statistics for EAG data.

Compound	Groups	df	F	p-value	sig
Beta ocimene	Dose	2	33.4	1.20E-12	yes
	HB	1	1.9	1.68E-01	no
	Side	1	4.3	4.04E-02	yes
	Dose × HB	2	3.4	3.46E-02	yes
	Dose × Side	2	3.6	2.86E-02	yes
	HB × Side	1	4.8	3.08E-02	yes
	Dose × HB × Side	2	0.6	5.42E-01	no
2-pentanone	Dose	2	17.3	1.73E-07	yes
	HB	1	0.3	5.74E-01	no
	Side	1	7.1	8.53E-03	yes
	Dose × HB	2	1.6	2.11E-01	no
	Dose × Side	2	1.3	2.70E-01	no
	HB × Side	1	1.5	2.24E-01	no
	Dose × HB × Side	2	1.0	3.71E-01	no
Phenethyl acetate	Side	1	3.5	6.83E-02	no
	HB	1	1.5	2.28E-01	no
	Side × HB	1	36.2	2.73E-07	yes

**Table 3:** Replicates for EAG data.

Dose	HB	Side	n
High	High	Left	10
High	High	Right	14
High	Low	Left	14
High	Low	Right	15
Low	High	Left	10
Low	High	Right	14
Low	Low	Left	14
Low	Low	Right	15
Med	High	Left	10
Med	High	Right	14
Med	Low	Left	14
Med	Low	Right	15

**Table 4:** FKB scores.

Colony ID	Score 1	Score 2	Average	HB Category	Experiment	
12	100	97.9	99.0	High	Proteomics	
6	100	97.1	98.5	High		
233	100	98.5	99.2	High		
365	93.8	96.9	95.3	High		EAG
8	100	100	100	High		
600	58.6	70.1	64.4	Low		
1010	39.3	57.8	48.6	Low		
530	49.3	58.3	53.8	Low		

## References

[b1] Cox-Foster D. L. . A metagenomic survey of microbes in honey bee colony collapse disorder. Science 318, 283–287 (2007). 17823314 10.1126/science.1146498

[b2] Gilliam M., Taber S. III. & Richardson G. V. Hygienic behavior of honey bees in relation to chalkbrood disease. Apidologie 14, 29–39 (1983).

[b3] Spivak M. & Gilliam M. Hygienic behaviour of honey bees and its application for control of brood diseases and Varroa: Part II. Studies on hygienic behaviour since the Rothenbuhler era. Bee world 79, 169–186 (1998).

[b4] Spivak M. & Gilliam M. Hygienic behaviour of honey bees and its application for control of brood diseases and varroa: Part I. Hygienic behaviour and resistance to American foulbrood. Bee World 79, 124–134 (1998).

[b5] Spivak M. Honey bee hygienic behavior and defense against Varroa jacobsoni. Apidologie 27, 245–260 (1996).

[b6] Spivak M. & Reuter G. Resistance to American foulbrood disease by honey bee colonies Apis mellifera bred for hygienic behavior. Apidologie 32, 555–565 (2001).

[b7] Spivak M. & Downey D. L. Field assays for hygienic behavior in honey bees (Hymenoptera: Apidae). Journal of economic entomology 91, 64–70 (1998).

[b8] Gramacho K. P. & Spivak M. Differences in olfactory sensitivity and behavioral responses among honey bees bred for hygienic behavior. Behavioral Ecology and Sociobiology 54, 472–479 (2003).

[b9] Guarna M. M. . A search for protein biomarkers links olfactory signal transduction to social immunity. BMC genomics 16, 1 (2015). 25757461 10.1186/s12864-014-1193-6 PMC4342888

[b10] Masterman R., Ross R., Mesce K. & Spivak M. Olfactory and behavioral response thresholds to odors of diseased brood differ between hygienic and non-hygienic honey bees (Apis mellifera L.). Journal of Comparative Physiology A 187, 441–452 (2001). 10.1007/s003590100216 11548991

[b11] Spivak M., Masterman R., Ross R. & Mesce K. A. Hygienic behavior in the honey bee (Apis mellifera L.) and the modulatory role of octopamine. Journal of neurobiology 55, 341–354 (2003). 12717703 10.1002/neu.10219

[b12] Swanson J. A. . Odorants that induce hygienic behavior in honeybees: identification of volatile compounds in chalkbrood-infected honeybee larvae. Journal of chemical ecology 35, 1108–1116 (2009). 19816752 10.1007/s10886-009-9683-8

[b13] Mondet F. . Antennae hold a key to Varroa-sensitive hygiene behaviour in honey bees. Scientific reports 5, 10454 (2015). 26000641 10.1038/srep10454 PMC4441115

[b14] Chakroborty N. K., Bienefeld K. & Menzel R. Odor learning and odor discrimination of bees selected for enhanced hygienic behavior. Apidologie 46, 499–514 (2015).

[b15] Sandoz J. C. Behavioral and neurophysiological study of olfactory perception and learning in honeybees. Front Syst Neurosci 5, 98, doi: 10.3389/fnsys.2011.00098 (2011). 22163215 PMC3233682

[b16] Hu H. . Proteome analysis of the hemolymph, mushroom body, and antenna provides novel insight into honeybee resistance against Varroa infestation. Journal of Proteome Research (2016). 10.1021/acs.jproteome.6b00423 27384112

[b17] Parker R. . Correlation of proteome-wide changes with social immunity behaviors provides insight into resistance to the parasitic mite, Varroa destructor, in the honey bee (Apis mellifera). Genome biology 13, 1 (2012). 10.1186/gb-2012-13-9-r81 PMC3491398 23021491

[b18] Guarna M. M. . Expression biomarkers used for the selective breeding of complex polygenic traits. BioRxiv, 10.1101/076174 (2016). PMC5566959 28827652

[b19] Nazzi F., Della Vedova G. & D’Agaro M. A semiochemical from brood cells infested by Varroa destructor triggers hygienic behaviour in Apis mellifera. Apidologie 35, 65–70 (2004).

[b20] Martin C. . Potential mechanism for detection by Apis mellifera of the parasitic mite Varroa destructor inside sealed brood cells. Physiological Entomology 27, 175–188 (2002).

[b21] Annoscia D., Del Piccolo F. & Nazzi F. How does the mite Varroa destructor kill the honeybee Apis mellifera? Alteration of cuticular hydrcarbons and water loss in infested honeybees. Journal of insect physiology 58, 1548–1555 (2012). 23041382 10.1016/j.jinsphys.2012.09.008

[b22] Salvy M. . Modifications of the cuticular hydrocarbon profile of Apis mellifera worker bees in the presence of the ectoparasitic mite Varroa jacobsoni in brood cells. Parasitology 122, 145–159 (2001). 11272645 10.1017/s0031182001007181

[b23] Mondet F. . Specific Cues Associated With Honey Bee Social Defence against Varroa destructor Infested Brood. Scientific reports 6 (2016). 10.1038/srep25444 PMC4853723 27140530

[b24] He X. J. . Starving honey bee (Apis mellifera) larvae signal pheromonally to worker bees. Scientific reports 6 (2016). 10.1038/srep22359 PMC4770327 26924295

[b25] Frasnelli E., Anfora G., Trona F., Tessarolo F. & Vallortigara G. Morpho-functional asymmetry of the olfactory receptors of the honeybee (Apis mellifera). Behav Brain Res 209, 221–225, doi: 10.1016/j.bbr.2010.01.046 (2010). 20138089

[b26] Anfora G., Frasnelli E., Maccagnani B., Rogers L. J. & Vallortigara G. Behavioural and electrophysiological lateralization in a social (Apis mellifera) but not in a non-social (Osmia cornuta) species of bee. Behavioural Brain Research 206, 236–239, doi: 10.1016/j.bbr.2009.09.023 (2010). 19766143

[b27] Kundu S., Ganguly A., Chakraborty T. S., Kumar A. & Siddiqi O. Synergism and Combinatorial Coding for Binary Odor Mixture Perception in Drosophila. eNeuro 3, doi: 10.1523/ENEURO.0056-14.2016 (2016). PMC4994066 27588303

[b28] Krofczik S., Menzel R. & Nawrot M. P. Rapid odor processing in the honeybee antennal lobe network. Front Comput Neurosci 2, 9, doi: 10.3389/neuro.10.009.2008 (2008). 19221584 PMC2636688

[b29] Reinhard J., Sinclair M., Srinivasan M. V. & Claudianos C. Honeybees learn odour mixtures via a selection of key odorants. PLoS One 5, e9110, doi: 10.1371/journal.pone.0009110 (2010). 20161714 PMC2817008

[b30] Deisig N., Giurfa M. & Sandoz J. C. Antennal lobe processing increases separability of odor mixture representations in the honeybee. J Neurophysiol 103, 2185–2194, doi: 10.1152/jn.00342.2009 (2010). 20181736

[b31] McAfee A. . Toward an Upgraded Honey Bee (Apis mellifera L.) Genome Annotation Using Proteogenomics. J Proteome Res 15, 411–421, doi: 10.1021/acs.jproteome.5b00589 (2016). 26718741

[b32] Gordon D. M. Dependence of necrophoric response to oleic acid on social context in the ant, Pogonomyrmex badius. Journal of chemical ecology 9, 105–111 (1983). 24408623 10.1007/BF00987774

[b33] Wilson E., Durlach N. & Roth L. Chemical releasers of necrophoric behavior in ants. Psyche 65, 108–114 (1958).

[b34] Carroll M. J. & Duehl A. J. Collection of volatiles from honeybee larvae and adults enclosed on brood frames. Apidologie 43, 715–730 (2012).

[b35] Maisonnasse A., Lenoir J.-C., Beslay D., Crauser D. & Le Conte Y. E-β-ocimene, a volatile brood pheromone involved in social regulation in the honey bee colony (Apis mellifera). PLoS One 5, e13531 (2010). 21042405 10.1371/journal.pone.0013531 PMC2958837

[b36] Maisonnasse A. . A scientific note on E-$\ beta $-ocimene, a new volatile primer pheromone that inhibits worker ovary development in honey bees. Apidologie 40, 562–564 (2009).

[b37] Trhlin M. & Rajchard J. Chemical communication in the honeybee (Apis mellifera L.): a review. Vet. Med 56, 265–273 (2011).

[b38] Traynor K. S., Le Conte Y. & Page R. E. Age matters: pheromone profiles of larvae differentially influence foraging behaviour in the honeybee, Apis mellifera. Animal Behaviour 99, 1–8 (2015).25580017

[b39] Rogers L. J., Rigosi E., Frasnelli E. & Vallortigara G. A right antenna for social behaviour in honeybees. Sci Rep 3, 2045, doi: 10.1038/srep02045 (2013). 23807465 PMC3694496

[b40] Rogers L. J. & Vallortigara G. From antenna to antenna: lateral shift of olfactory memory recall by honeybees. PLoS One 3, e2340, doi: 10.1371/journal.pone.0002340 (2008). 18523636 PMC2394662

[b41] Villa J. D., Danka R. G. & Harris J. W. Simplified methods of evaluating colonies for levels of Varroa Sensitive Hygiene (VSH). Journal of apicultural research 48, 162–167 (2009).

[b42] Le Conte Y. . Varroa destructor changes its cuticular hydrocarbons to mimic new hosts. Biol Lett 11, 20150233, doi: 10.1098/rsbl.2015.0233 (2015). 26041867 PMC4528474

[b43] Panasiuk B., Skowronek W., Bienkowska M., Gerula D. & Wegrzynovicz P. Age of worker bees performing hygienic behaviour in a honeybee colony. Journal of Apicultural Science 54, 109–115 (2010).

[b44] Olsson S. B. & Hansson B. S. Electroantennogram and single sensillum recording in insect antennae. Methods Mol Biol 1068, 157–177, doi: 10.1007/978-1-62703-619-1_11 (2013). 24014360

[b45] Humphrey S. J., Azimifar S. B. & Mann M. High-throughput phosphoproteomics reveals *in vivo* insulin signaling dynamics. Nat Biotechnol 33, 990–995, doi: 10.1038/nbt.3327 (2015). 26280412

