# Supplementary material for: Odorant cues linked to social immunity induce lateralized antenna stimulation in honey bees (Apis mellifera L.)
Source: Sci Rep. 2017 Apr 7;7:46171. doi: 10.1038/srep46171 (PMC5384011; doi:10.1038/srep46171)
Supplement: Supplementary Information [file srep46171-s1.pdf]

Odorant cues linked to social immunity induce lateralized antenna stimulation in honey bees (*Apis mellifera* L.)

Alison McAfee, Troy F. Collins, Lufiani L. Madilao, Leonard J. Foster

Table of Contents

Supplementary Figure S1	p. 2-5
Supplementary Figure S2	p. 6
Supplementary Figure S3	p. 7
Supplementary Figure S4	p. 8

B-ocimene

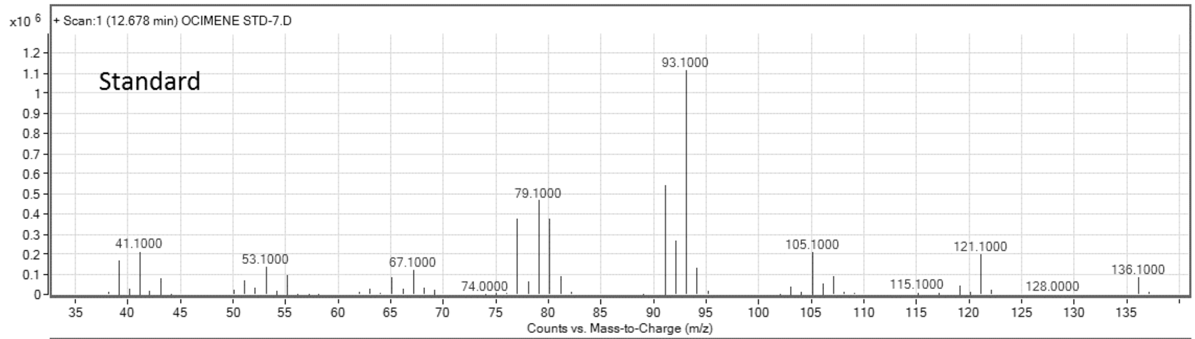

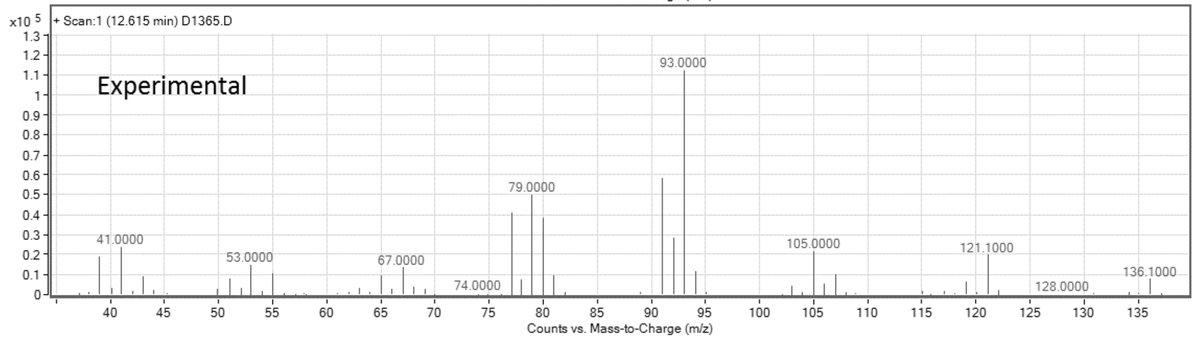

2-pentanone

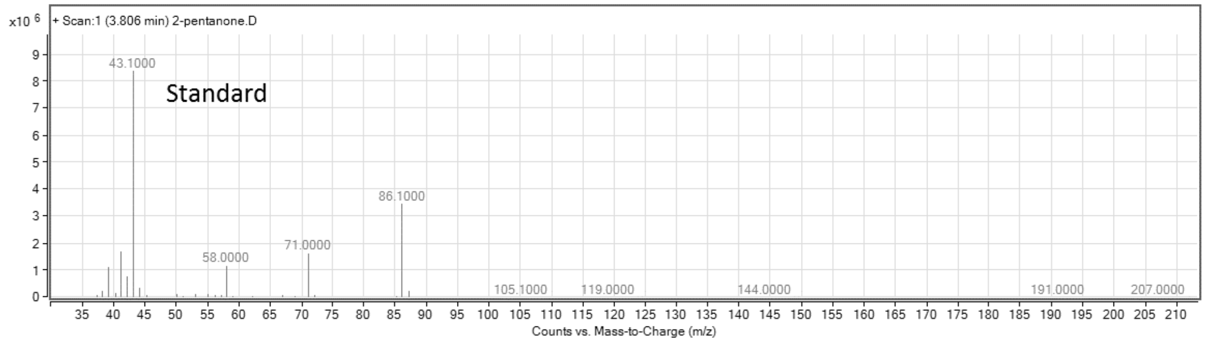

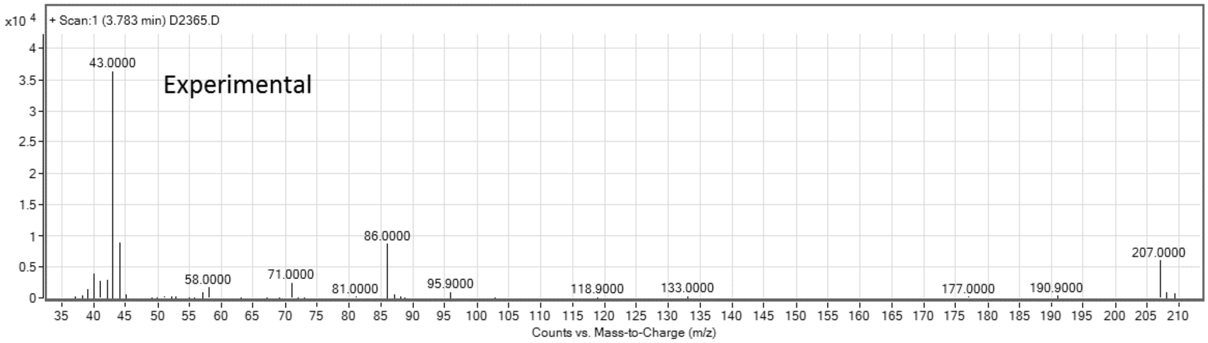

2-propanol

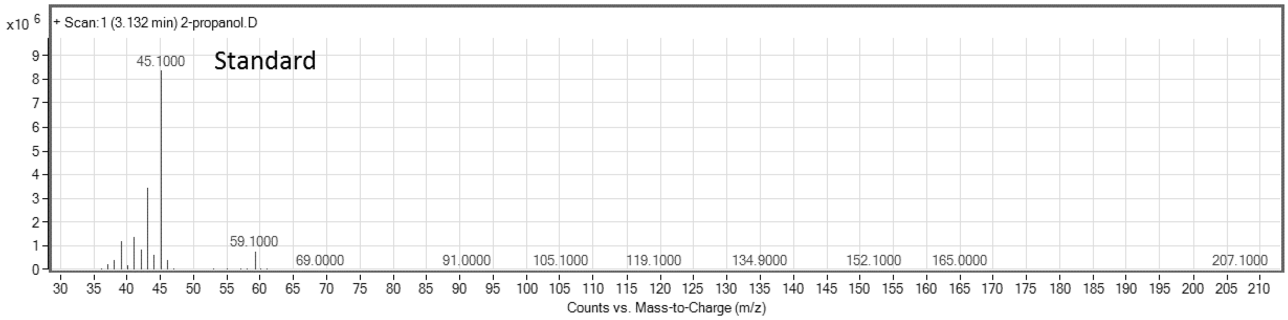

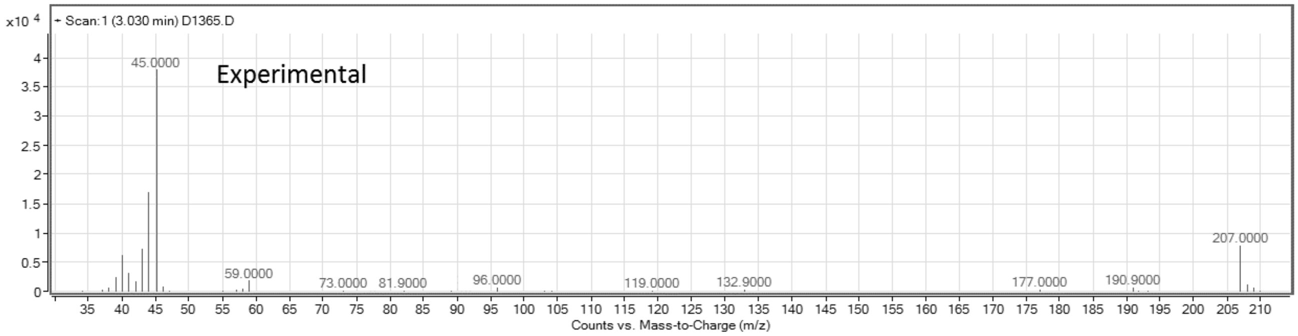

Oleic acid

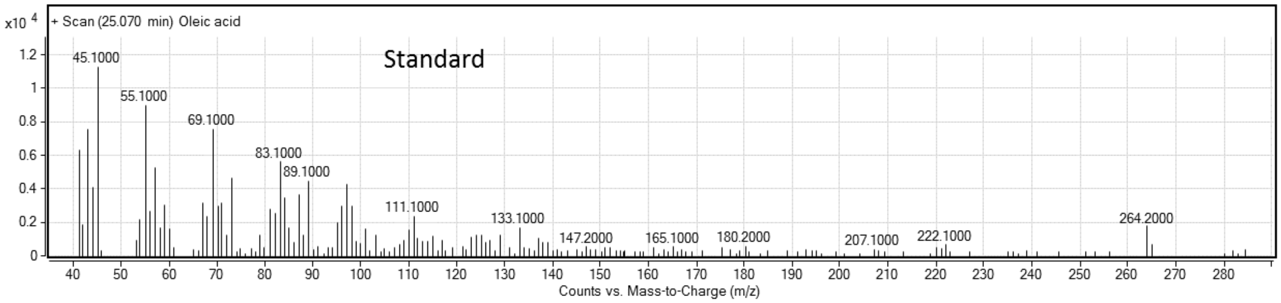

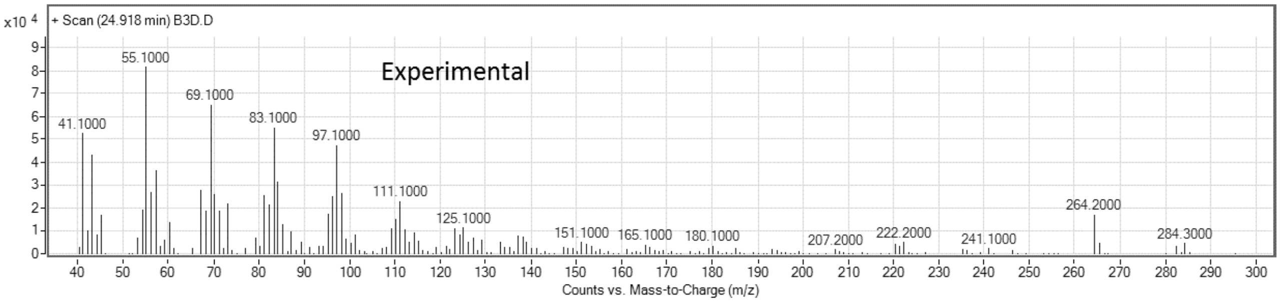

A-thujene

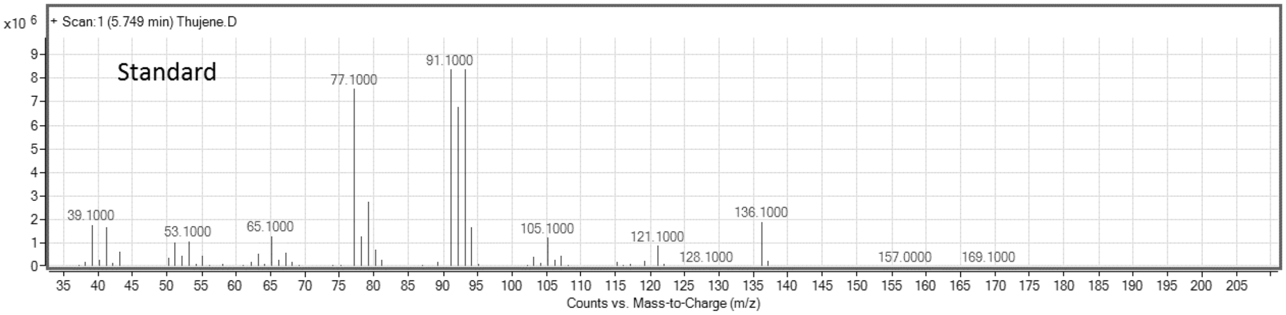

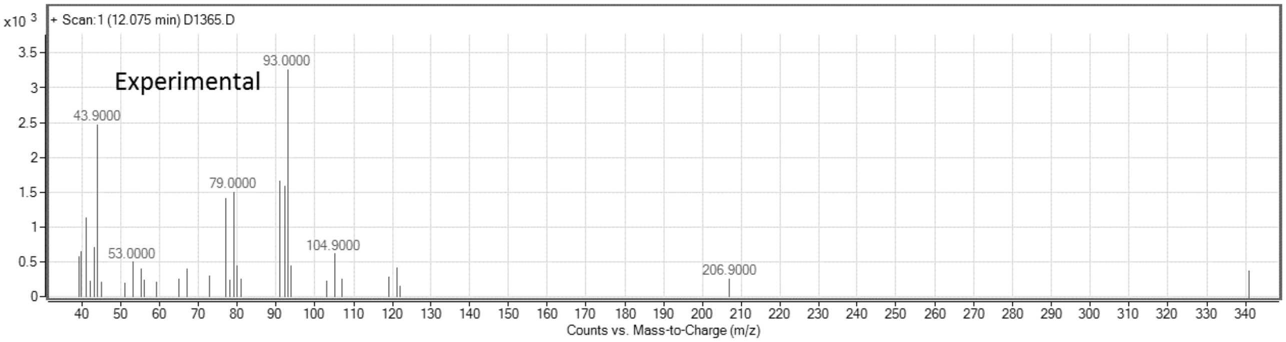

2-3-butanediol

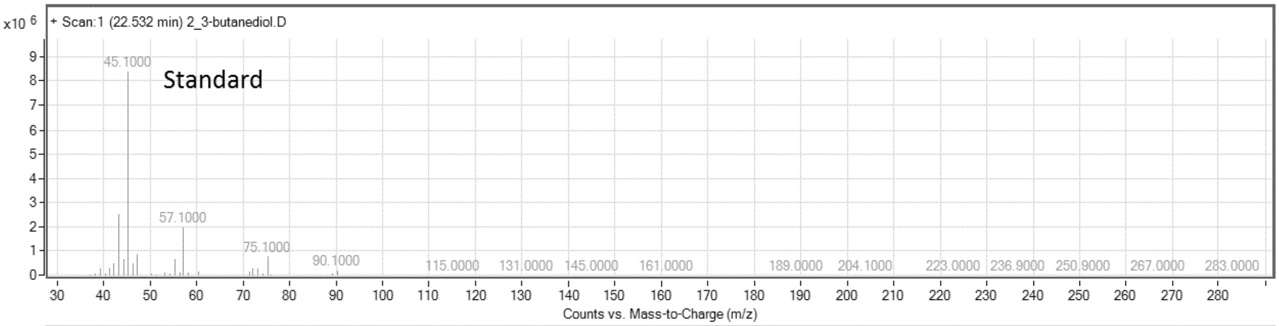

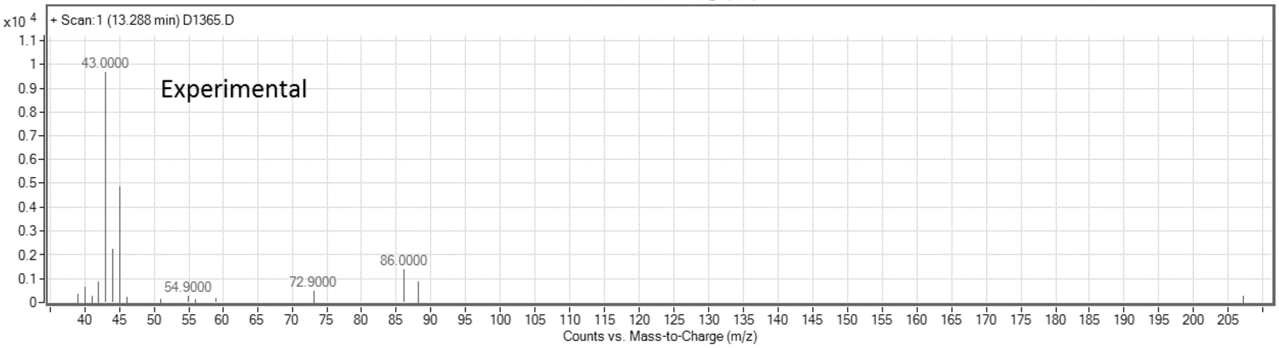

A-pinene

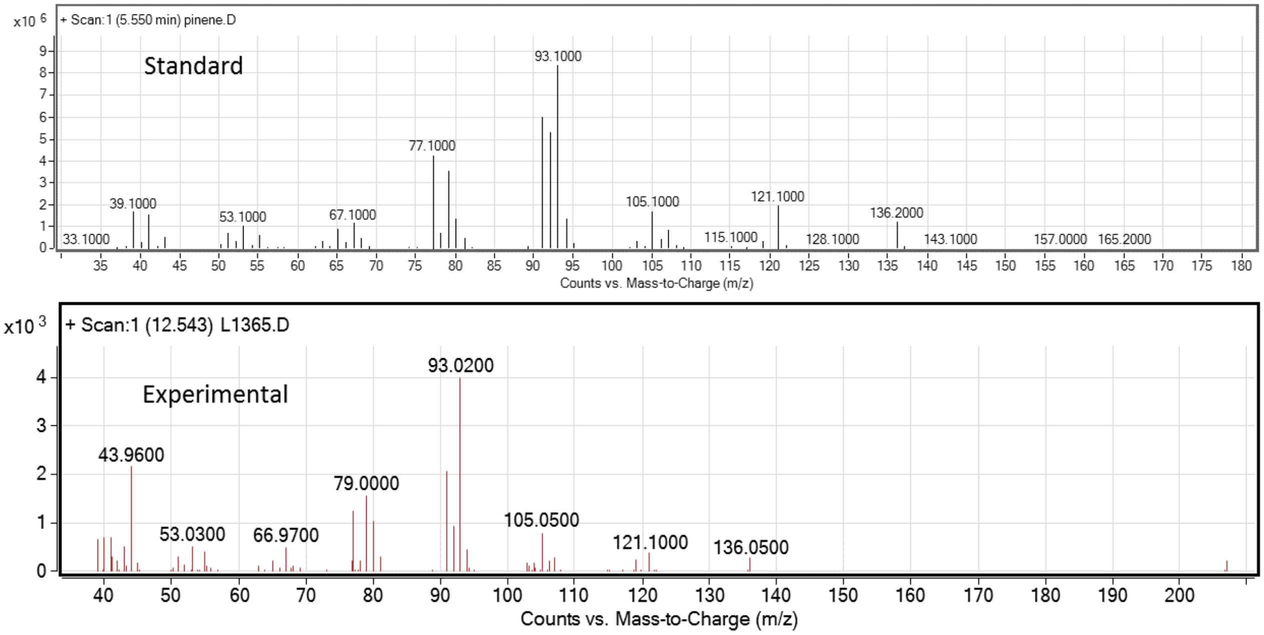

Carbon dioxide

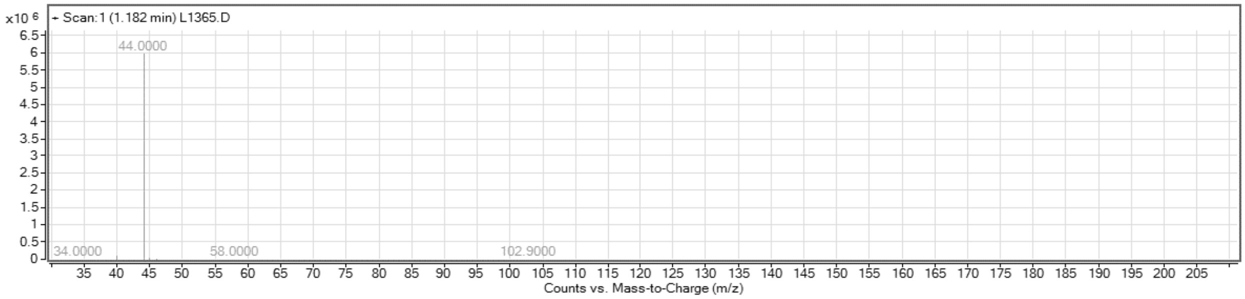

2-methyl tetradecane

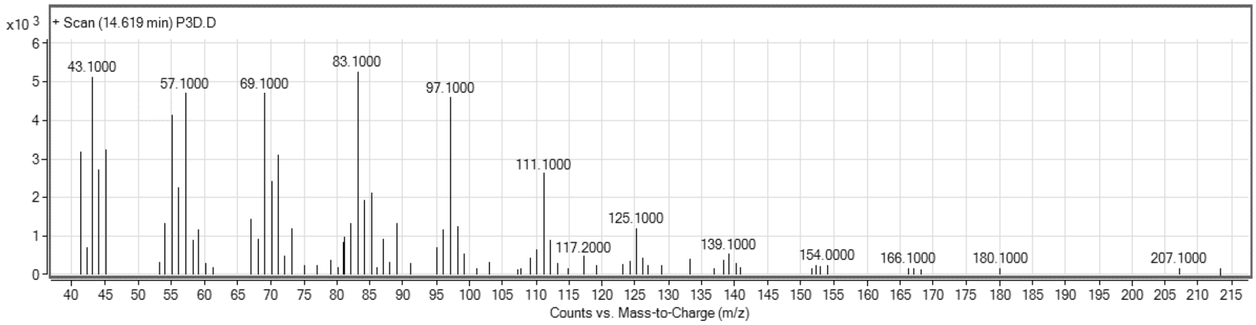

Supplementary Figure S1. Example spectra of differentially emitted compounds compared to standards. Only β-ocimene, 2-propanol, 2-pentanone and oleic acid matched both retention times and fragment spectra to the synthetic standards. All others either did not match or a synthetic standard was not available. 2-methyl tetradecane and oleic acid were run on an Agilent 6890N/5975C Inert XL MSD and all others were run on an Agilent 7890A/5975C Inert XL MSD. For more details see Methods, subheading: FKB GC-MS Sample Collection.

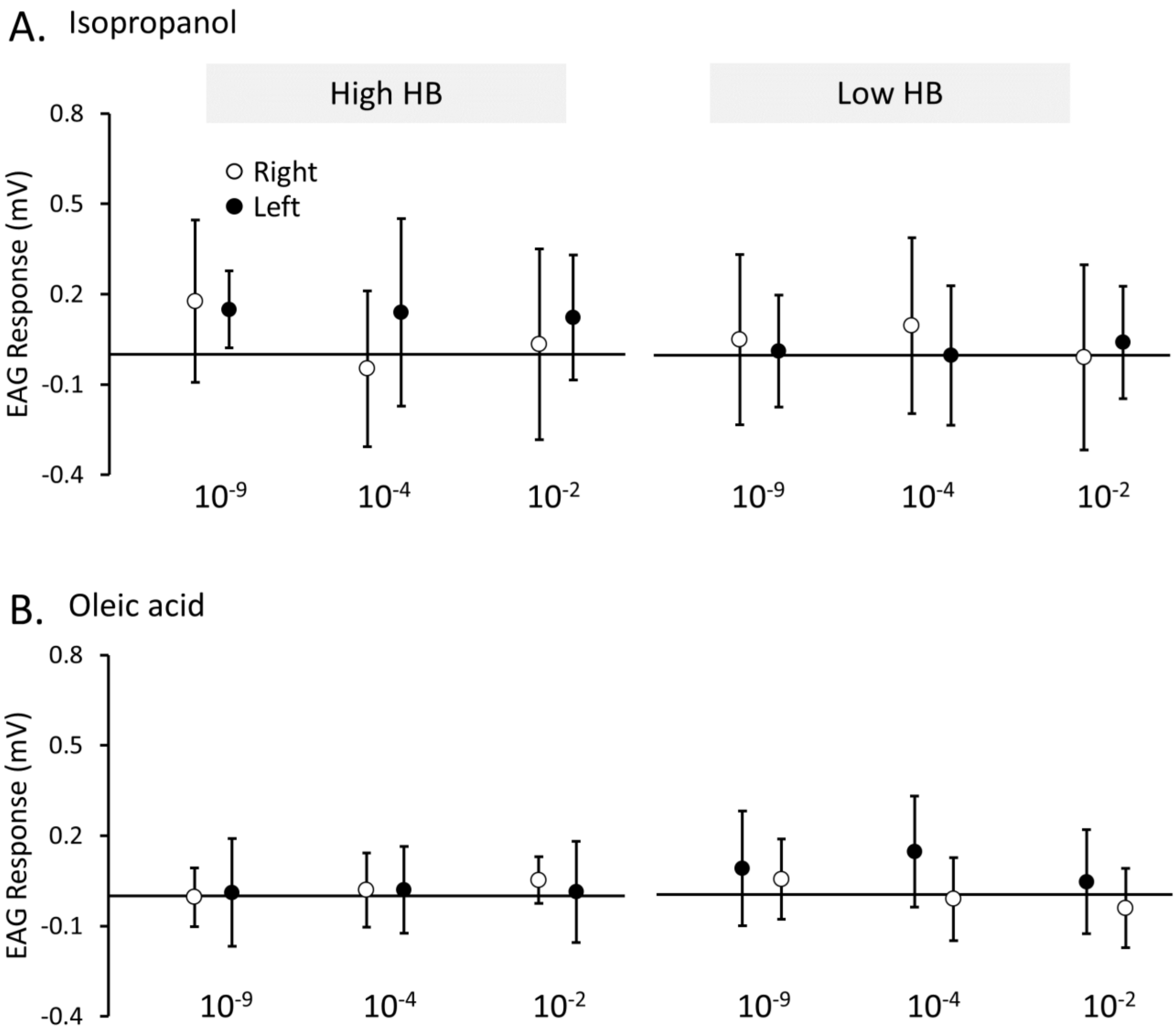

Figure S2. *Electroantennographic data for oleic acid and isopropanol stimulations.* Doses were applied at three dilutions (10^{-9}, 10^{-4} and 10^{-2} v/v). The background solvent stimulus was subtracted from the solvent + compound stimulus to yield the mV stimulation of the compound alone. No significant differences between dose, side or HB were found with these compounds (three-factor ANOVA).

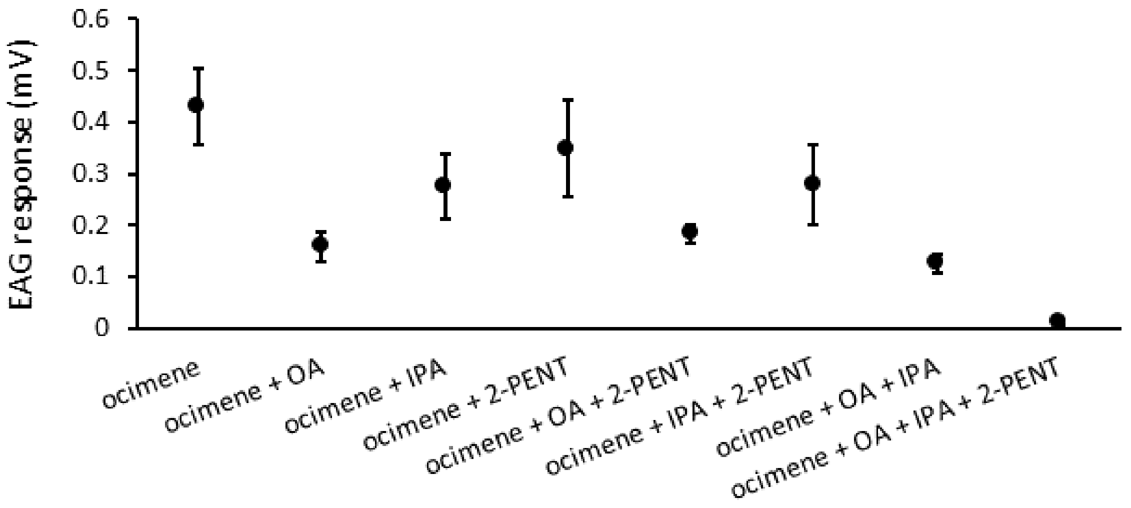

Supplementary Figure S3. *Systematic combinatorial analysis of disease odors.* All combinations of oleic acid (OA), isopropanol (IPA), 2-pentanone (2-PENT) and β-ocimene were mixed to 1% solutions (v/v) and used to stimulate honey bee worker antennae. No stimulations produced stronger EAG responses than β-ocimene (mixtures without β-ocimene are not shown). Error bars represent variance.

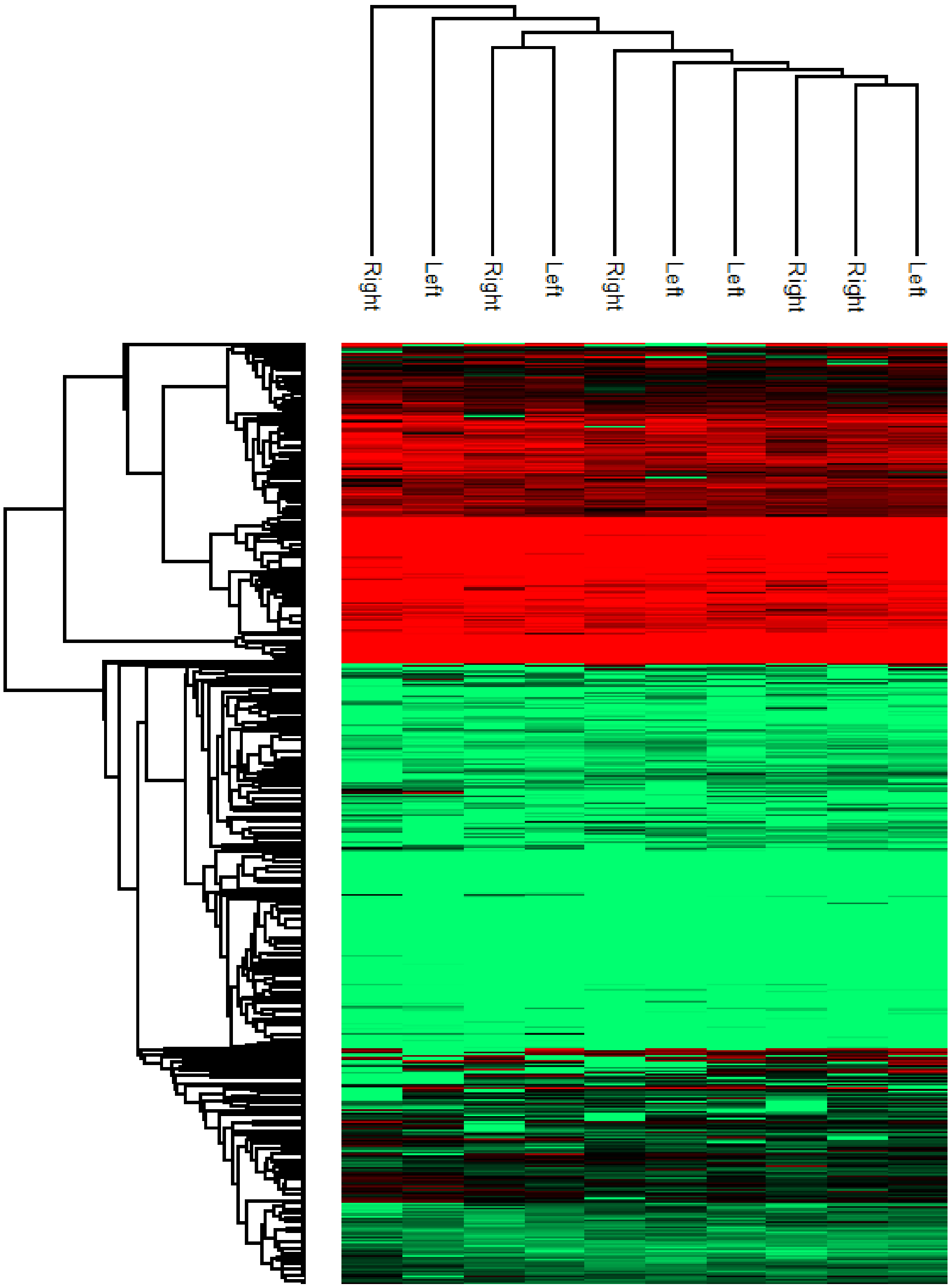

Supplementary Figure S4. *Proteomic analysis of left and right antennae from hygienic bees.* Label-free quantitation (LFQ) was performed on left and right antennae from five hygienic colonies. No significantly different proteins were identified.
